# Early-life stress perturbs the epigenetics of *Cd36* concurrent with adult onset of NAFLD in mice

**DOI:** 10.1038/s41390-023-02714-y

**Published:** 2023-07-21

**Authors:** Qi Fu, Jenna M. Frick, Maura F. O’Neil, Olivia C. Eller, E. Matthew Morris, John P. Thyfault, Julie A. Christianson, Robert H. Lane

**Affiliations:** 1 Department of Research Administration, Children’s Mercy Hospital, Kansas City, MO USA; 2 Department of Anatomy and Cell Biology, School of Medicine, University of Kansas Medical Center, Kansas City, KS USA; 3 Department of Pathology and Laboratory Medicine, School of Medicine, University of Kansas Medical Center, Kansas City, KS USA; 4 Department of Molecular and Integrative Physiology, School of Medicine, University of Kansas Medical Center, Kansas City, KS USA; 5 Research Service, Kansas City VA Medical Center, Kansas City, KS USA; 6 Department of Administration, Children’s Mercy Hospital, Kansas City, MO USA

## Abstract

**Background:**

Non-alcoholic fatty liver disease (NAFLD) is one of the most common liver diseases in the U.S. and worldwide. The roles of early postnatal life stress (EPLS) and the fatty acid translocase (CD36) on the pathogenesis of adult-onset NAFLD remain unknown. We hypothesized that EPLS, in the form of neonatal maternal separation (NMS), would predispose mice towards developing adult NAFLD, increase hepatic CD36 expression, and differentially methylate *Cd36* promoter concurrently.

**Methods:**

NMS was performed on mice from postnatal day 1 to 21 and a high-fat/high-sucrose (HFS) diet was started at 4 weeks of age to generate four experimental groups: Naive-control diet (CD), Naive-HFS, NMS-CD, and NMS-HFS.

**Results:**

NMS alone caused NAFLD in adult male mice at 25 weeks of age. The effects of NMS and HFS were generally additive in terms of NAFLD, hepatic *Cd36* mRNA levels, and hepatic *Cd36* promoter DNA hypomethylation. *Cd36* promoter methylation negatively correlated with *Cd36* mRNA levels. Two differentially methylated regions (DMRs) within *Cd36* promoter regions appeared to be vulnerable to NMS in the mouse.

**Conclusions:**

Our findings suggest that NMS increases the risk of an individual, particularly male, towards NAFLD when faced with a HFS diet later in life.

**Impact:**

- The key message of this article is that neonatal maternal separation and a postweaning high-fat/high-sucrose diet increased the risk of an individual, particularly male, towards NAFLD in adult life.
- What this study adds to the existing literature includes the identification of two vulnerable differentially methylated regions in hepatic Cd36 promoters whose methylation levels very strongly negatively correlated with Cd36 mRNA.
- The impact of this article is that it provides an early-life environment-responsive gene/promoter methylation model and an animal model for furthering the mechanistic study on how the insults in early-life environment are “transmitted” into adulthood and caused NAFLD.

## Introduction

Experiencing stress or adversity during early development increases the likelihood of developing chronic health disorders such as obesity^1–3^ and insulin resistance^4,5^ later in adulthood in both humans and animal models. As a result, a plethora of studies now exist exploring how early-life stress initiates the pathogenesis of obesity and insulin resistance. In contrast, the influence of early life stress on the pathogenesis of non-alcoholic fatty liver disease (NAFLD) remains largely unknown and receives relatively little attention.

NAFLD stands as the most common liver disease in the United States and worldwide. NAFLD is characterized primarily by excessive triglycerides (TG) in hepatocytes.^6–8^ The pathogenesis of NAFLD is multifactorial and remain elusive. Studies in recent years suggest that fatty acid translocase or cluster of differentiation 36 (CD36) plays a causal role in the pathogenesis of NAFLD.

CD36 is a multifunctional membrane receptor involved in long-chain fatty acid uptake, lipid metabolism, and inflammation.^9–12^ Hepatocytes normally express low levels of CD36 though CD36 expression increases with lipid-rich diets in humans and mouse models.^13–18^ Upregulation of CD36 membrane protein in liver elevates cellular uptake of fatty acids and positively correlates with hepatic steatosis.^14,19–21^ Liver-specific CD36 knockout attenuates steatosis in mouse models of NAFLD.^22,23^

Previously, our group demonstrated that an adverse early-life environment consisting of late pregnancy maternal stress and a maternal high-fat/high-sucrose (HFS) diet upregulated hepatic CD36 in a mouse model of adult NAFLD. The NAFLD occurred predominantly in male offspring, and a postweaning HFS diet further exacerbated the hepatic steatosis and upregulation of CD36.^24^ The increases in CD36 expression in this model occurred within the context of differentially methylated regions of the *Cd36* promoter that correlated directly with hepatic NAFLD.^24^ A question exists as to whether other mouse models of early-life stress exposure will induce *Cd36* promoter differential DNA CpG methylation, CD36 upregulation, and adult NAFLD, and, if so, this would suggest a potentially common conserved mechanism for the pathogenesis of NAFLD.

To answer this question, we used a previously established mouse model of neonatal maternal separation (NMS), that demonstrates increased susceptibility to weight gain, particularly on a HFS diet.^5^ We hypothesized that NMS mice would have greater incidence of NAFLD in adulthood. We also hypothesized that these phenotypical changes would be associated with *Cd36* promoter differential DNA CpG methylation and CD36 upregulation.

## Methods

### Animals

All animal procedures were approved by the University of Kansas Medical Center Institutional Animal Care and Use Committee in compliance with the National Institute of Health Guide for the Care and Use of Laboratory Animals. Male and female C57Bl/6 mice (Charles River, Wilmington, MA) were housed in the Research Support Facility at the University of Kansas Medical Center. Mice were housed at 22 °C on a 12-h light cycle (600–1800) and received water and food ad libitum.

The NMS mouse model has been published preciously,^5,25^ with some modifications in this study. Briefly, pregnant C57Bl/6 dams were delivered to the animal facility between 14 and 16 days of gestation. Litters were divided equally into NMS and naive groups. From postnatal day (P) 1 until P21, all pups from a single cohort of pregnant dams were used for this study. NMS pups were removed en masse and placed in a clean glass beaker with bedding from their home cage for 180 min (11 a.m.–2 p.m.). The beaker was placed in an incubator maintained at 33 °C and 50% humidity. Naive mice remained undisturbed in their home cage except for normal animal husbandry. All mice were weaned on P22 and pair-housed with same sex litter mates and ad libitum access to water and a control diet (CD) composed of 20% kcal protein, 70% kcal carbohydrate (3.5% sucrose), and 10% kcal fat (Research Diets, Inc. New Brunswick, NJ, Cat. No. D12110704).

At 4 weeks of age, half of the naive and NMS groups were randomly placed on a high-fat/high-sucrose (HFS) diet, consisting of 20% kcal protein, 35% kcal carbohydrate (15% sucrose), and 45% kcal fat (4.73 kcal/g; Research Diets Cat. No. D12451, Supplementary Table 1) to mimic the higher fat and simple sugar content in a western-style diet.

At week 25 of life, mice were overdosed with inhaled isoflurane (>5%). Liver was dissected and weighed. Half of the liver was flash frozen in liquid nitrogen and stored at −80 °C. Half was fixed in 10% formalin.

### Hepatic histology

Formalin-fixed livers were paraffin embedded. Slices (4 µm) were stained by hematoxylin and eosin and Masson’s trichrome. The NAFLD activity score (NAS) was evaluated by a pathologist blinded to the experimental groups by using the Kleiner scoring system.^26^ Briefly, the score is defined as the unweighted sum of the scores for steatosis (0–3), lobular inflammation (0–3), and ballooning (0–2). Fibrosis was recorded separately. A minimum of 5% steatosis (NAS score 1) was used for the operational minimal definition of histological NAFLD. NAS score 1–2 were largely considered mild NAFLD and considered “not nonalcoholic steatohepatitis (NASH)”. A score of ≥5 is interpreted as NASH.^26^

### Hepatic TG quantification

Liver tissues were ground in liquid nitrogen. A portion of the grinds was weighed followed by TG isolation and quantification using Triglyceride Quantification Kit (MAK266, Sigma-Aldrich) following the manufacturer’s manual. The hepatic TG levels were expressed as mg/g of liver tissue.

### Membrane protein extraction and immunoblotting

Liver membrane protein was extracted using a Mem-PER Plus Membrane Protein Extraction Kit (Thermo Scientific) and quantified using a Pierce BCA Protein Assay Kit (Thermo Scientific). Quantification of CD36 membrane protein was done using capillary immunoassay using Wes Simple Western system (Proteinsimple) as described previously.^24^ 1:100 dilution of anti-CD36 antibody (ab133625, abcam) was used. 1:2000 pan Cadherin (ab51043, abcam) was used as an internal control.

### RNA isolation and real-time reverse transcriptase (RT)–PCR

Total RNA isolation was performed by using RNeasy Mini Kit (74904, Qiagen) following the manufacturer’s instructions, including DNase I treatment. RNA was quantified spectrophotometrically. The integrity of RNA was assessed with an Agilent 2100 bioanalyzer in conjunction with the RNA 6000 Nano kit (Agilent). cDNA was synthesized using a High-capacity cDNA Reverse Transcription Kit (4368814, Thermo Fisher Scientific). Real-time RT-PCR was performed as described earlier.^24^ Target primers and probes for *Cd36* total mRNA, *Cd36* transcripts initiated from promoter 1 (P1 transcripts) and promoter 2 (P2 transcripts) were described previously.^24^ Transcripts initiated from promoter 3 (P3 transcripts) were calculated by subtracting P1 and P2 transcripts from total *Cd36* since no specific assays can be designed for P3 transcripts.^24^ Primer efficiencies for these three sets of *Cd36* primers/probes were tested and calculated from the slope using the formula Efficiency (%) = (10^–1/slope^ − 1) × 100. Peptidylprolyl isomerase A (Ppia) was chosen as an internal control after assessing Ppia, glyceraldehyde-3-phosphate dehydrogenase (Gapdh), beta-actin (Actb), beta-2 microglobulin (B2m), beta-glucuronidase (Gusb), hydroxymethylbilane synthase (Hmbs), and hypoxanthine guanine phosphoribosyl transferase (Hprt) as candidate housekeeping genes. PCR conditions and calculations of mRNA expression were performed as demonstrated previously.^24^

### Bisulfite pyrosequencing

Genomic DNA was isolated from liquid nitrogen-ground liver powder by using the DNeasy Blood & Tissue Kit (Qiagen) including RNase treatment. DNA quantity and purity were estimated spectrophotometrically. Bisulfite treatment of genomic DNA was performed using an EpiTect Plus Bisulfite Kit (Qiagen) as instructed in the manual.

For each PCR, bisulfite-treated DNA equivalent to 20 ng of the DNA prior to bisulfite treatment was used. Primers for PCR and sequencing were designed by using PyroMark Assay Design 2.0 software (Qiagen). Primer sequences, PCR conditions, and pyrosequencing for the three promoters were published previously.^24^ Promoter 2 has total 7 CpG sites within the proximal promoter. No specific assays can be designed for CpGs (-558, -556, and -241). Besides CpGs (-845, -740) studied previously, CpGs (-664, -645) were also added to this study by using the forward primer 5’TGAGTGAATAGAGAGATTGTTGTGGGATA and the reverse primer 5’Biosg/ACACACACCCCAAAAACAAA. PCR condition was 95 °C for 10 min, followed by 50 cycles of 94 °C for 30 s, 62 °C for 30 s, and 72 °C for 30 s. Pyrosequencing was performed using Q48 Autoprep (Qiagen) with sequencing primer 5’AGATTGTTGTGGGATAT.

### Statistics

Statistical analysis was performed using GraphPad Prism 8 software (GraphPad Software). Chi-square tests were performed to evaluate the diagnosis and severity of NAFLD between groups. To evaluate the severity of NAFLD, Chi-square tests were performed by subcategorizing NAS scores into NAS 0 (no steatosis), NAS 1–2 (steatosis, no NASH), NAS 3–4 (possible NASH or NASH), and NAS 5 and above (NASH) for evaluating the severity of NAFLD. Data are expressed as mean ± standard deviation (SD). Data were analyzed for normality or lognormality first and then analyzed by using 2-way ANOVA to assess main and interaction effects, with NMS and diet as independent variables. When significant main or interaction effect was detected, Bonferroni post hoc testing was used to identify the means that differ. An additive effect was reported when significant *p* values of both NMS-HFS compared with NMS-CD and NMS-HFS compared with Naive-HFS were found, but *p* interaction was not significant.^24,27^ Correlation analyses were computed using Pearson correlation coefficients. The level of significance was set at *p* < 0.05 for all statistical tests.

## Results

### NMS and HFS significantly increased the body fat percentage

Both NMS and HFS significantly increased body fat percentage in both male (Supplementary Fig. 1) and female mice^28^ at 14 and 25 weeks of life. In this model, NMS significantly decreased serum corticosterone (CORT) in males^29^ and resulted a trend toward increased serum CORT in female mice.^28^

### NMS and HFS significantly increased the prevalence and severity of hepatic steatosis in adult mice

**Fig. 1:**
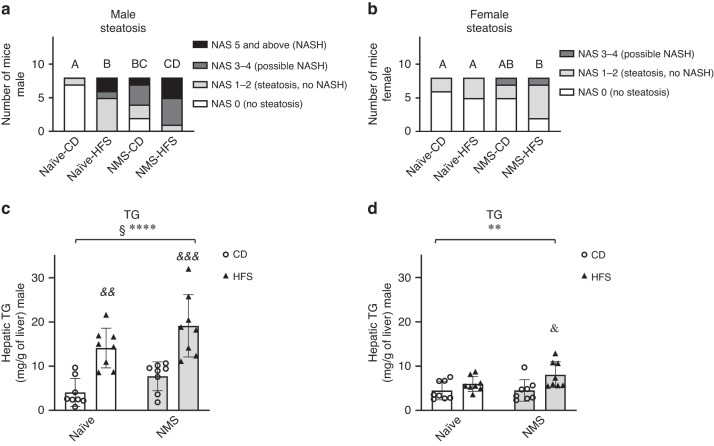
NMS and HFS increased the prevalence and severity of hepatic steatosis in adult mice in a sex-specific manner. NAFLD activity scores in male (**a**) and female (**b**) mice. Labeled groups without a common letter differ (steatosis severity A < B < C < D), *p* < 0.05. Hepatic TG levels in male (**c**) and female (**d**) mice. Values are means ± SDs. *n* = 8. § and * denote significant NMS and diet effects, respectively. &, &&, &&& *p* < 0.05, 0.01, 0.001 HFS vs CD. CD control diet, HFS high-fat/high-sucrose diet, Naive no stress control group, NMS neonatal maternal separation, NAS NAFLD activity score, TG triglycerides.

Hepatic steatosis was diagnosed based on NAS scores, with the criteria of 5% steatosis as the operational minimal definition of histological NAFLD.^26^ In males, Chi-square analysis revealed that all the three experimental groups had significantly more hepatic steatosis diagnosed compared to Naive-CD mice (Naive-HFS: *p* = 0.0004, NMS-CD: *p* = 0.0117, NMS-HFS: *p* = 0.0004) (Fig. 1a). In females, NMS-HFS mice had a significantly higher incidence of steatosis compared with Naive-CD (*p* = 0.0455) and Naive-HFS mice (*p* = 0.0455), but not with NMS-CD mice (*p* = 0.0721) (Fig. 1b). Only a few mild fibrosis cases were observed among the groups with no statistical significance detected (data not shown).

Next, the impact of NMS and HFS diet on the severity of steatosis was determined. Chi-square analyses were performed by subcategorizing NAS scores into NAS 0 (no steatosis), NAS 1–2 (steatosis, no NASH), NAS 3–4 (possible NASH or NASH), and NAS 5 and above (NASH).^26^ In males, NMS-CD mice had more severe steatosis than Naive-CD mice (*p* = 0.0209) and NMS-HFS mice had significantly more cases of severe steatosis than Naive-HFS mice (*p* = 0.0389) (Fig. 1a). These data indicated that NMS significantly worsened the development of severe NAFLD in male mice, regardless of diet. However, in females, most of the observed steatosis was mild (only 2 with NAS 3–4 and no NAS 5) and only NMS-HFS mice had more severe steatosis compared to Naive-CD (*p* = 0.0455) or Naive-HFS mice (*p* = 0.0455) (Fig. 1b).

To further confirm these histological findings, hepatic TG content was quantified. Both NMS and HFS diet had significant main effects on increasing hepatic TG contents in male livers (*p* = 0.0154 and *p* < 0.0001, respectively) (Fig. 1c). Only a significant diet effect was found to increase hepatic TG levels in females (*p* = 0.0047) (Fig. 1d).

### HFS diet significantly upregulated hepatic CD36 membrane protein in both sexes

**Fig. 2:**
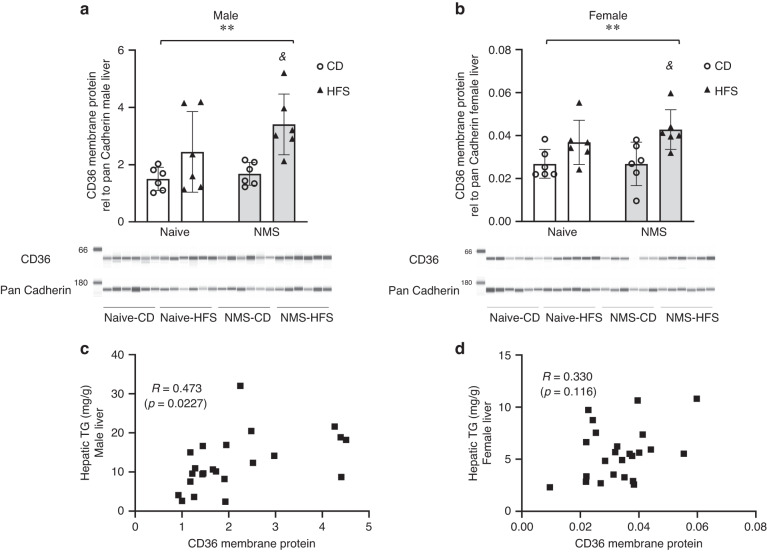
HFS diet significantly upregulated hepatic CD36 membrane protein in both sexes. CD36 membrane protein expression and immunoblot image (**a**, **b**) and correlation analysis (**c**, **d**) in livers of 25-week-old male and female mice from the Naive and NMS groups and on the postweaning diet of CD and HFS. **a**, **b** Values are means ± SDs. *n* = 8. *Denotes significant diet effect. ^&^*p* < 0.05 HFS vs CD. CD control diet, HFS high-fat/high-sucrose diet, Naive no stress control group, NMS neonatal maternal separation, *R* Pearson’s *r*, TG triglycerides.

HFS diet had a significant effect on increasing hepatic CD36 membrane protein levels in male and female mice (*p* = 0.0021 and 0.0024, respectively) (Fig. 2a, b). NMS did not significantly impact hepatic CD36 membrane protein levels in either sex (*p* > 0.05); however, NMS-HFS mice had significantly higher CD36 membrane protein levels compared to NMS-CD in both male and female mice (*p* = 0.025 and 0.0405, respectively) (Fig. 2a, b).

A significant positive correlation was found between the levels of CD36 membrane protein and hepatic TG contents in male mice (*p* = 0.0227) (Fig. 2c), with a similar trend in female mice (*p* = 0.116) (Fig. 2d).

### NMS and HFS diet synergistically upregulated *Cd36* total mRNA and P2-initiated transcripts in male livers

Three different promoters ((P)1, P2, or P3) initiate transcription of the *Cd36* gene, with P3 serving as the primary promoter under “normal” conditions.^24^ To determine whether NMS or HFS diet impacted transcriptional initiation of *Cd36*, specific primers/probes were designed for RT-PCR.^24^ The assay efficiencies for the total *Cd36* mRNA, P2 transcripts, and P1 transcripts were 91.89%, 90.84%, and 91.75%, respectively, allowing for comparisons of the impact of NMS and HFS on the transcriptional activity between promoters.

**Fig. 3:**
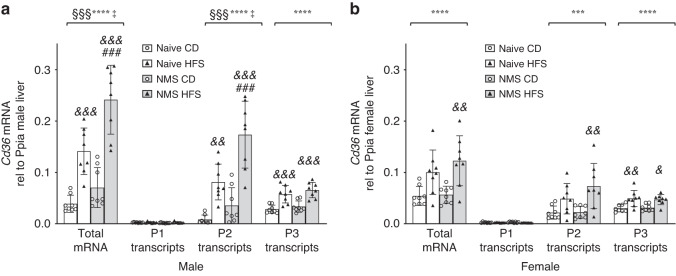
NMS and HFS diet synergistically upregulated *Cd36* total mRNA and P2-initiated transcripts in male livers. Hepatic mRNA expression of *Cd36* total mRNA, P1, P2, and P3 transcripts in male (**a**) and female (**b**) livers of 25-week-old mice from the Naive and NMS groups and on the postweaning diet of CD and HFS. Values are mRNA expression levels relative to internal control Ppia and expressed as means ± SDs. *n* = 8. §, *, and ‡ denote significant effects of NMS, diet, and a NMS/diet interaction, respectively. &, &&, &&& *p* < 0.05, 0.01, 0.001 HFS vs CD. ### *p* < 0.001 NMS-HFS vs Naive-HFS. CD control diet, HFS high-fat/high-sucrose diet, Naive no stress control group, NMS neonatal maternal separation, P promoter.

As expected, in the male Naive-CD mice, P3-initiated transcripts were the major mRNA variant species in the liver (Fig. 3a and Supplementary Fig. 2a). However, P3- and P2-initiated transcripts were similarly expressed in female Naive-CD livers (Fig. 3b and Supplementary Fig. 3a). P1 transcripts were negligibly expressed in both sexes indicating that promoter 1 is minimally used for transcription in mouse livers (Fig. 3), which is consistent with our previous data.^24^

In male mice, both NMS and HFS diet had significant main effects on the upregulation of *Cd36* total mRNA (*p* < 0.0001 and *p* = 0.0003, respectively) and P2 transcripts (*p* < 0.0001 and *p* = 0.0003, respectively) (Fig. 3a). Importantly, NMS and HFS diet synergistically upregulated both *Cd36* total mRNA and P2 transcripts (*p* = 0.0417 and 0.0333, respectively) with significantly higher levels in NMS-HFS mice compared to Naive-HFS mice (*p* = 0.0009 and 0.0007, respectively). Only a significant effect of HFS diet was found on P3 transcripts upregulation (*p* < 0.0001) (Fig. 3a). No significant difference was found for P1 transcripts between groups (Fig. 3a). Strikingly, in comparison to Naive-CD male livers, NMS-HFS increased the expression of P2 transcripts by 21.63-folds (0.173 ± 0.065 vs 0.008 ± 0.009), of P3 by 2.36-folds (0.066 ± 0.015 vs 0.028 ± 0.008), and of total *Cd36* mRNA by 6.21-folds (0.242 ± 0.067 vs 0.039 ± 0.017). Altogether, NMS and HFS diet shifted the major promoter from P3 to P2 in male livers (Fig. 3a and Supplementary Fig. 2b).

In female mice, only a significant effect of HFS diet was found on the upregulation of total *Cd36* mRNA (*p* < 0.0001), P2-initiated transcripts (*p* = 0.0005), and P3 transcripts (*p* < 0.0001) (Fig. 3b). Relative to Naive-CD mice, exposure to NMS and HFS diet increased the expression of P2 transcripts by 3.39-folds (0.073 ± 0.044 vs 0.022 ± 0.013), of P3 transcripts by 1.56-folds (0.047 ± 0.010 vs 0.030 ± 0.007), and of total *Cd36* mRNA by 2.26-folds (0.123 ± 0.049 vs 0.054 ± 0.019). Both P2 and P3 were the main transcription initiating promoters without significant difference between the two (Fig. 3b and Supplementary Fig. 3a, b).

### *Cd36* total mRNA, P2- and P3-initiated transcripts were significantly positively correlated with the hepatic TG contents

**Fig. 4:**
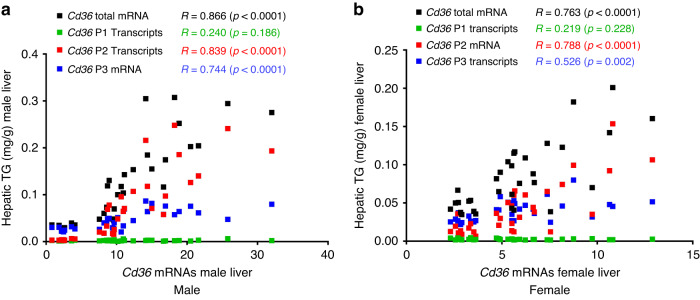
*Cd36* total mRNA, P2- and P3-initiated transcripts were significantly positively correlated with the hepatic TG contents. Correlations between hepatic TG contents and *Cd36* total mRNA, P1, P2, and P3 transcripts in 25-week-old male (**a**) and female (**b**) mice from the Naive and NMS groups and on the postweaning diet of CD or HFS. CD control diet, HFS high-fat/high-sucrose diet, Naive no stress control group, NMS neonatal maternal separation, *R* Pearson’s *r*, TG triglycerides.

Expression levels of *Cd36* total mRNA and the P2 and P3 transcripts were correlated with hepatic TG contents. As with the protein expression data, the *Cd36* total mRNA, P2 transcripts, and P3 transcripts were significantly positively correlated with the hepatic TG contents in both male and female livers (Fig. 4). P1 transcript levels were not correlated with hepatic TG (Fig. 4).

### NMS and HFS significantly hypomethylated promoters 2 and 3 of *Cd36* in male liver

**Fig. 5:**
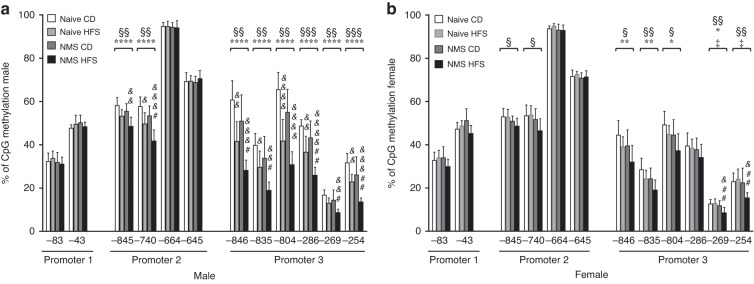
NMS and HFS diet significantly hypomethylated promoters 2 and 3 of *Cd36* in male liver. Percent of DNA CpG methylation of *Cd36* promoters 1, 2, and 3 in the livers of 25-week-old male (**a**) and female (**b**) mice from the Naive and NMS groups and on the postweaning diet of CD or HFS. The negative number below each CpG site indicates the number of base pair upstream relative to the transcription start site of the corresponding promoter, respectively. Values are means ± SDs. *n* = 8. §, *, and ‡ denote significant effects of NMS, diet, and a NMS/diet interaction, respectively. &, &&, &&&, &&&& *p* < 0.05, 0.01, 0.001, 0.0001 HFS vs CD. #, ## *p* < 0.05, 0.01 NMS-HFS vs Naive-HFS. CD control diet, HFS high-fat/high-sucrose diet, Naive no stress control group, NMS neonatal maternal separation.

DNA hypomethylation has previously been associated with transcriptional upregulation of hepatic *Cd36* promoters.^24^ To determine if NMS and/or HFS diet-increased expression of P2 and P3 was related to DNA hypomethylation, bisulfite pyrosequencing of all three promoter regions was carried out. As expected, in the male livers, both NMS and HFS diet had significant main effects on the hypomethylation of two (CpGs (-845) and (-740)) out of four CpG sites studied around P2 and all six CpG sites around P3 (Fig. 5a). Importantly, additive effects of NMS and HFS diet were found on the hypomethylation of CpG (-740) in P2 and five (CpGs (-846, -835, -286, -269, -254)) out of the six CpG sites in P3, with NMS-HFS mice having significantly lower methylation compared to either Naive-HFS or NMS-CD mice (*p* < 0.05) (Fig. 5a). We defined the regions around CpG (-845, -740) of P2 and the regions around all the six CpG sites as differentially methylated regions (DMRs), which were sensitive to both NMS and postweaning HFS diet.

Paralleling the more moderate impacts of NMS and HFS diet on the expression of *Cd36* mRNA, female liver exhibited less DNA hypomethylation, compared to the male mice (Fig. 5b). Only a NMS effect was observed for two (CpG (-845) and (-740)) of the four CpGs in P2 (*p* = 0.0132 and 0.0136, respectively) (Fig. 5b). NMS and HFS diet had significant main effects on the hypomethylation of five (-846, -835, −804, -269, -254) out of the six CpGs in P3. A synergistic effect between NMS and HFS was found on CpG (-269) and CpG (-254) (*p* = 0.0266 and 0.0197, respectively) (Fig. 5b). Altogether, the environment sensitive DMRs identified in male livers were similarly impacted by NMS and HFS diet in female livers, although to a lesser extent.

### *Cd36* total mRNA, P2- and P3-initiated transcripts were strongly and negatively correlated with promoter methylation in a CpG- and RNA variant-specific manner

**Fig. 6:**
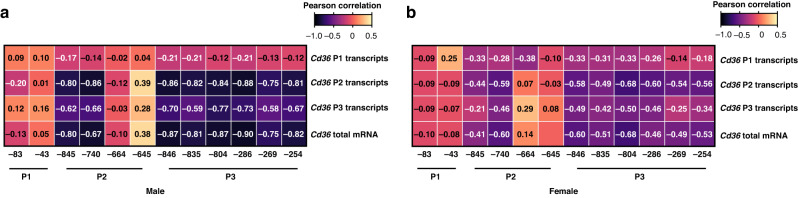
*Cd36* total mRNA, P2- and P3-initiated transcripts were strongly and negatively correlated with promoter methylation in a CpG- and RNA variant-specific manner. Pearson correlation score obtained from comparisons of promoter CpG methylation with *Cd36* total mRNA and promoter transcripts in male (**a**) and female (**b**) livers at 25 weeks of age. The negative number below the graphs indicates the number of base pair upstream relative to the transcription start site of the corresponding exon, respectively. Values are Pearson’s *r*. P promoter.

Correlation analysis revealed strong negative correlations between the methylation of promoters and the levels of *Cd36* total mRNA or variant transcripts in both sexes (Fig. 6). Specifically, CpG (-845) and (-740) of P2 and all six CpGs of P3 were very strongly and negatively correlated with total *Cd36* mRNA and P2 transcripts, and moderately, with P3 transcripts (Fig. 6 and Supplementary Table 2). Importantly, these correlations were CpG site specific because the methylation status of the two studied CpGs in P1 and CpG (-664) and (-645) in P2 was not correlated with expression of any mRNA species of *Cd36*. Similarly, these correlations were *Cd36* mRNA variant specific because the levels of P1-initiated transcripts were not correlated with methylation of any CpG site tested across the three promoters.

These results, together with our previous findings in an independently conducted study in a mouse model of different early life environment (Table 1),^24^ indicated that the DMRs in P2 and P3 have conserved transcriptional regulatory roles in mouse liver that are sensitive to early life environments.

**Table 1:** Comparison of our two mouse models of early-life environment-related NAFLD in adulthood.

Mouse model	AME^a^	NMS^a^				
Early-life environment	Maternal HFS diet (5 weeks pre-pregnancy through lactation) and maternal life stress (the last third pregnancy period)	Neonatal maternal separation from postnatal day 1 through day 21; No maternal diet manipulation				
Research group handled the animals	Medical College of Wisconsin ((Lane, RH)	University of Kansas (Christianson, JA)				
Animal species/strain	C57BL/6J (The Jackson Laboratory)	C57BL/6 (Charles River)				
Animal sex	Male only	Male and female				
Animal age at end of study	17 weeks of life	25 weeks of life				
Age when postweaning diet started	3 weeks of life	4 weeks of life				
Postweaning diet	CD	HFS	CD	HFS		
Fat, % in kcal	10%	40%	10%	45%		
Carbohydrate (Sucrose), % in kcal	73% (0%)	43% (15%)	70% (3.5%)	35% (29.1%)		
Protein, % in kcal	17%	17%	20%	20%		
Cholesterol, wt/wt	0	0.15%	0	0		
Sex (Experimental group–Postweaning diet)	Male (AME-CD)	Male (AME-HFS)	Male (NMS-CD)	Female (NMS-CD)	Male (NMS-HFS)	Female (NMS-HFS)
Hepatic steatosis	Mild steatosis^c^	Moderate steatosis^b,c^	Mild steatosis^c^	NS	Moderate steatosis and NASH^c^	Mild steatosis^c^
CD36 Membrane protein	↑^d^	↑^b,c,d,e^	NS	NS	↑^b,e^	↑^b,e^
CD36 total mRNA expression	↑^d^	↑^b,c,d,e^	↑^d^	NS	↑^b,c^	↑^b,e^
CD36 P1-initiated transcripts	NS	NS	NS	NS	NS	NS
CD36 P2-initiated transcripts	↑^c,d^	↑^b,c,d,e^	↑^d^	NS	↑^b,c,d,e^	↑^b,e^
CD36 P3-initiated transcripts	↑^d^	↑^b,c,d,e^	NS	NS	↑^b,e^	↑^b,e^
P1 CpG (-83, -43) methylation	NS	NS	NS	NS	NS	NS
P2 CpG (-845) methylation	↓^d^	↓^b,d,e^	↓^d^	↓^d^	↓^b,d,e^	↓^d^
P2 CpG (-740) methylation	↓^d^	↓^b,d,e^	↓^d^	↓^d^	↓^b,c,d,e^	↓^b,d^
P2 CpG (-664, -645) methylation	Not tested	Not tested	NS	NS	NS	NS
P3 CpG (-846) methylation	↓^c,d^	↓^b,c,d,e^	↓^d^	↓^d^	↓^b,c,d,e^	↓^d,e^
P3 CpG (-835) methylation	↓^c,d^	↓^b,c,d,e^	↓^d^	↓^d^	↓^b,c,d,e^	↓^d,e^
P3 CpG (-804) methylation	↓^c,d^	↓^b,c,d,e^	↓^d^	↓^d^	↓^b,d,e^	↓^d,e^
P3 CpG (-286) methylation	↓^d^	↓^b,d,e^	↓^d^	NS	↓^b,c,d,e^	NS
P3 CpG (-269) methylation	↓^d^	↓^d,e^	↓^d^	↓^d^	↓^b,c,d,e^	↓^b,c,d,e^
P3 CpG (-254) methylation	↓^d^	↓^d,e^	↓^d^	↓^d^	↓^b,c,d,e^	↓^b,c,d^

*AME* adverse maternal environment, *CD* control diet, *HFS* high-fat/high-sucrose diet, *NAFLD* non-alcoholic fatty liver disease, *NASH* nonalcoholic steatohepatitis, *NMS* neonatal maternal separation, *NS* no significant statistical difference, *P1* promoter 1, *P2* promoter 2, *P3* promoter 3.

^a^There were four experimental groups of each sex for each animal model of AME or NMS. Listed in the table are only the two diet groups of the maternal experimental group (AME or NMS) for each model. The statistical differences labeled are comparisons within each model, not between the two mouse models.

^b^Significantly different from corresponding postweaning CD group of the same maternal group *p* < 0.05.

^c^Significantly different from corresponding control maternal group of the same diet, *p* < 0.05.

^d^Significant main effect of early-life environment, *p* < 0.05.

^e^Significant main effect of postweaning diet, *p* < 0.05.

## Discussion

The core findings of this study suggest that NMS in mice decreases DNA CpG methylation in the hepatic *Cd36* promoter, increases hepatic *Cd36* mRNA and protein levels, and causes NAFLD in adult male mice. Specific findings of this study include (1) the observation that postweaning HFS diet exacerbates the impact of NMS on NAFLD, and the putative associated mechanisms included in this study; (2) the positive correlation of *Cd36* mRNA and protein levels with hepatic TG content; and (3) the identification of two vulnerable DMRs in the *Cd36* promoter whose methylation levels very strongly negatively correlated with *Cd36* mRNA in a mRNA variant- and CpG site-specific manner. The findings in males parallel similar findings from a previous model involving a maternal adverse early-life environment conducted at another institution (Table 1). Considering the methodological differences between the two model, we speculate that hypomethylation of the hepatic *Cd36* DMRs represents a conserved mechanism through which adverse early life event and stressors initiate the pathogenesis of later life NAFLD.

A link between early life events and the development of NAFLD later in life does exist in the literature. For example, prenatal hypoxia followed by subsequent hypoxia at 6 months of life led to NALFD in male rats.^30^ This model of NAFLD is striking as it occurred in the absence of an obesogenic diet.^30^ Our previous study demonstrating a link between early life events and NAFLD described the impact of an adverse maternal environment (AME) consisting of both maternal chronic stress during late pregnancy, induced by non-invasive environmental perturbation such as reduced bedding, and maternal HFS diet from 5 weeks preconception through lactation.^24^ AME-exposed male offspring developed more frequent hepatic steatosis than the controls from this model despite consuming a low-fat control diet for 14 weeks after weaning. Interestingly, a postweaning HFS diet exacerbated the hepatic steatosis phenotype in the AME male offspring. In this previous study, the influence of prenatal stress on adult-onset NAFLD could not be determined because early-life exposure to obesogenic diet alone has been shown to cause the NAFLD phenotype.^31,32^ In contrast, the current study demonstrates that NMS alone increased the risk of developing NAFLD later in adulthood in a sex-specific manner.

A link between CD36 and adult NAFLD has been well-established in prior studies. Upregulation of CD36 membrane protein positively correlates with elevated hepatocyte uptake of fatty acids and hepatic steatosis in models of NAFLD originating in adulthood.^14,19,20^ Conversely, hepatocyte-specific deletion of CD36 attenuates steatosis in rodent models of adult NAFLD.^22,23,33^ Our previous study and the current data further support a role of CD36 in NAFLD development, as both showed a strong correlation between hepatic *Cd36* mRNA and protein levels with TG content, as well as increased steatosis. The similarities of the findings between the two disparate models of early life stress suggest that upregulation of CD36 may provide a conserved mechanism underlying the pathogenesis of NAFLD. Further work is needed to determine the necessity of CD36 in early life stress-mediated NAFLD development, particularly in the absence of a HFS diet.

Epigenetic modifications are also associated with early life stress exposure and the development of negative health outcomes.^34^ As such, the two models of early life stress appear to share a conserved mechanism of increasing CD36 through the hypomethylation of the differentially methylated regions (DMRs) of the hepatic *Cd36* promoters 2 and 3 (Table 1). Both P2 and P3 of *Cd36* function as active promoters that nature conserves between mouse and human.^22,35–38^ Literature focusing upon transcriptional regulation of P2 is rare. Multiple transcription factor response elements have been reported within promoter 3.^22,33,39,40^ These transcription factors include liver X receptor (LXR), prognane X receptor (PXR), peroxisome proliferator activated receptor (PPAR) gamma, and the aryl hydrocarbon receptor (AhR).^22,33,39,40^ These TFs bind to their cognate *cis*-regulatory elements around −230 bp for PPAR, −400bp for PXR, −1080 bp for LXR, and −1250 bp for AhR upstream of the P3 transcription start site (TSS), respectively.^22,33,39,40^ Binding of these transcription factors to their respective response elements upregulates total *Cd36* mRNA.

Functional studies of these two promoters suggest that they operate somewhat independently. For example, a diet containing a PPARα ligand upregulates hepatic P2-initiated transcripts, but not P3 transcripts.^36,37^ We specifically found that exposure to both NMS and HFS diet increased P2 transcripts approximately 22 (male) and 3.4 (female) fold, whereas increased P3 transcripts only 2.4 (male) and 1.6 (female) fold. Of importance, methylation levels of the identified DMRs in P2 and P3 significantly correlated with P2 transcript levels. These significant changes and correlations suggest that the methylation status of these DMRs are responsive to environmental exposures, such as stress and diet, and play a key role in the transcriptional regulation of hepatic *Cd36* gene.^41^ Further studies are needed to elucidate other pathways by which the early postnatal life environment mediate changes in *Cd36* gene expression and epigenetics, including how promoter 2 is activated.

Cd36 promoters 2 and 3 are CpG sparse with most of the CpG sites intermediately methylated. Genome-wide studies have shown that the genomic regions with intermediate DNA methylation (IM) is a conserved chromatin signature of genome regulation.^42^ These IM regions have average 57% methylation and are allele-independent. The IM signature enriches for regions with multiple indicators of regulatory functions, particularly those associated with enhancers, however, the precise function of intermediate DNA methylation states is not clear.^42^ Sae-Lee et al. have shown that the IM regions had binding specificity to certain DNMT isoforms over others using overexpression experiments in a human cell line.^43^ Importantly, DMRs in IM regions have been reported to be enriched among those associated with human diseases, such as allergic sensitization, in human association studies.^44^ Our methylation data of the mouse Cd36 promoters 2 and 3 had the feature of IM [except the CpGs (-664 and -665 of P2), which are inside the simple repeats and to be expected to be highly methylated] and responsive to early-life environments. Therefore, our animal/disease/gene model provides a unique opportunity to study epigenetic regulation of IM chromatin signature.

Consistent with human observations and previous rodent models, male mice in the study had more severe outcomes compared to the female mice.^45,46^ Both showed similar trends in terms of NAFLD prevalence/score, CD36 levels, and methylation changes, as well as hypomethylation at the same DMRs. Also, the severity of hepatic steatosis positively and significantly correlated with hepatic CD36 expression and negatively correlated with DNA CpG methylation of hepatic *Cd36* P2 and P3, suggesting a conserved mechanism between the sexes. Future work will be necessary to understand how sex is influencing the impact of early life stress and HFS diet on hepatic outcomes, particularly considering that previous studies of the same NMS model have shown similar changes in urogenital hypersensitivity and function in male and female mice.^47,48^

Caution is always necessary when attempting to apply data from mouse models to human pathophysiology. Studies have shown that the development and severity of induced NAFLD is dependent on strain and species of the mice.^49^ Further studies are needed to see if the findings in the current and the comparator studies are applicable to other species, especially in humans.

In summary, exposure to NMS in mice resulted in adult-onset NAFLD, concurrent with upregulation of hepatic CD36 expression and hypomethylation of *Cd36* promoter DMRs. The remarkable similarity of these findings to that of another model suggest that this may be a conserved response to early life stress. Regardless, these findings highlight the importance of the early postnatal life environment on the health in later life and provide a gene/animal model tool to further understanding the mechanisms at play.

### Supplementary information

Supplementary Figure 1

Supplementary Figure 2

Supplementary Figure 3

Supplementary Table 1

Supplementary Table 2

## Data Availability

The datasets generated during and/or analyzed during the current study are available from the corresponding author on reasonable request.
