# Supplementary material for: Early-life stress perturbs the epigenetics of Cd36 concurrent with adult onset of NAFLD in mice
Source: Pediatr Res. 2023 Jul 21;94(6):1942–50. doi: 10.1038/s41390-023-02714-y (PMC10665193; doi:10.1038/s41390-023-02714-y)
Supplement: Supplementary file 1 — Supplementary Figure 1 [file 41390_2023_2714_MOESM1_ESM.pdf]

Supplementary data

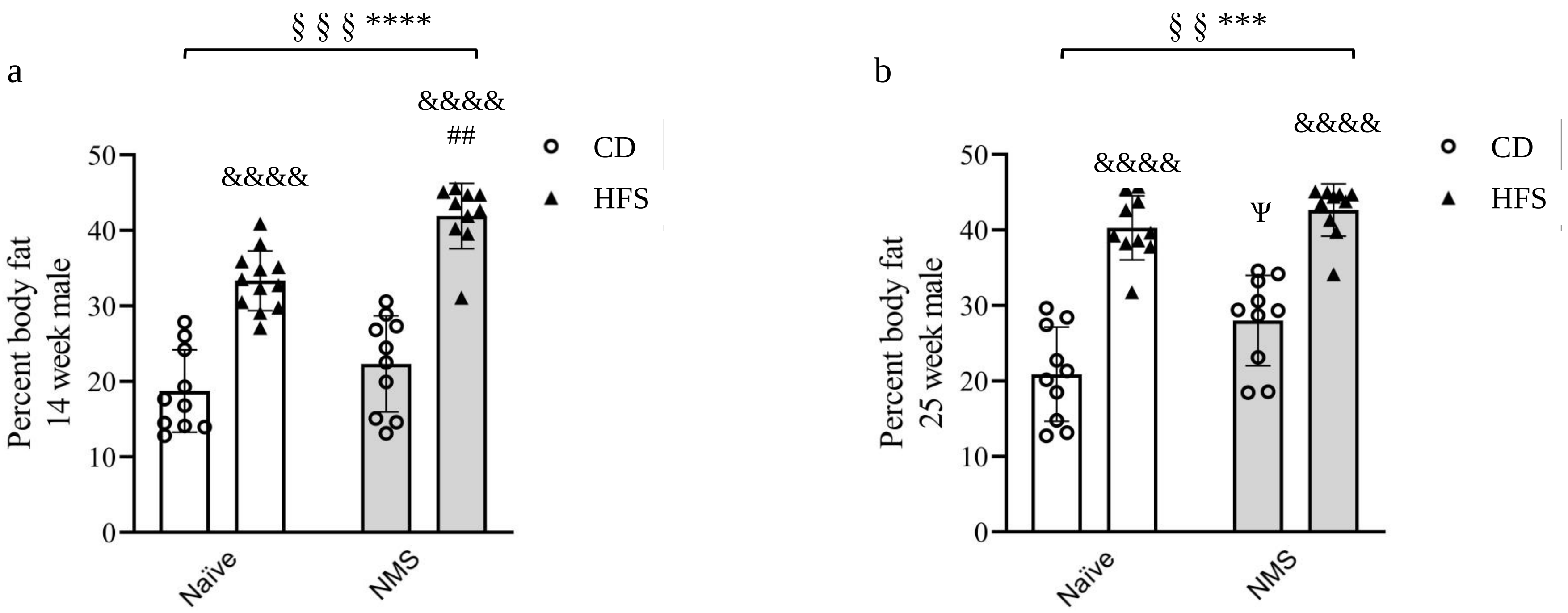

Supplementary Figure 1. Effects of NMS and HFS on body fat percentage at 14 (a) and 25 (b) weeks of life in male mice. Values are means ± SDs. n = 10 ~12. § and * denote significant effects of NMS and diet, respectively. &&&& p<0.0001 HFS vs CD; ## p<0.01 NMS-HFS vs Naïve-HFS; Ψ p<0.05 NMS-CD vs Naïve-CD. CD, control diet; HFS, high-fat/high-sucrose diet; Naïve, no stress control group; NMS, neonatal maternal separation.
