# Supplementary material for: Early-life stress perturbs the epigenetics of Cd36 concurrent with adult onset of NAFLD in mice
Source: Pediatr Res. 2023 Jul 21;94(6):1942–50. doi: 10.1038/s41390-023-02714-y (PMC10665193; doi:10.1038/s41390-023-02714-y)
Supplement: Supplementary file 3 — Supplementary Figure 3 [file 41390_2023_2714_MOESM3_ESM.pdf]

Supplementary data

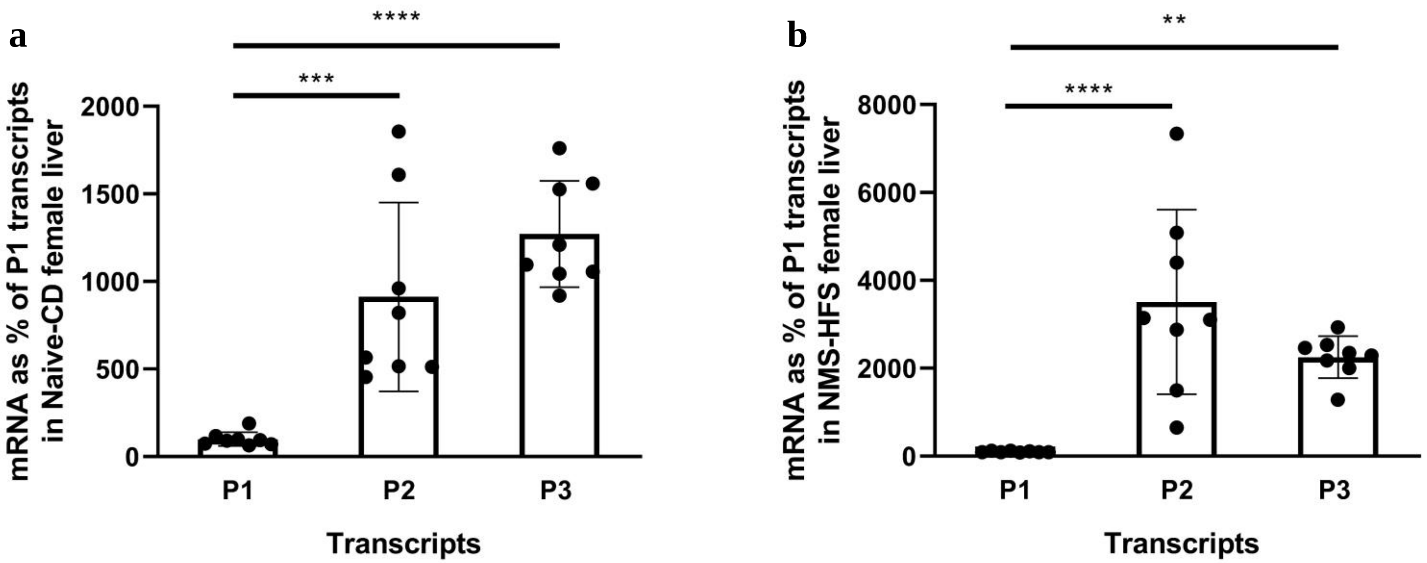

Supplementary Figure 3. Hepatic expression of Cd36 promoter-initiated transcripts as percent of P1 transcripts in NMS-HFS (a) and NMS-HFS (b) in female mice. Values are means ± SDs. n = 8. ** $p< 0.01$; *** $p<0.0001$; **** $p < 0.00001$.
