# Supplementary material for: Early-life stress perturbs the epigenetics of Cd36 concurrent with adult onset of NAFLD in mice
Source: Pediatr Res. 2023 Jul 21;94(6):1942–50. doi: 10.1038/s41390-023-02714-y (PMC10665193; doi:10.1038/s41390-023-02714-y)
Supplement: Supplementary file 4 — Supplementary Table 1 [file 41390_2023_2714_MOESM4_ESM.pdf]

Supplementary Table 1. Fatty acid profile of experimental diets

Diets	HFS*	CD#
Total Fatty Acid (g)	202.5	45
Fatty Acid	g	g
C10, Capric	0.1	0.0
C12, Lauric	0.2	0.0
C14, Myristic	2.1	0.3
C15	0.1	0.0
C16, Palmitic	36.8	6.4
C16:1, Palmitoleic, n=9	2.5	0.3
C17	0.7	0.1
C18, Stearic	19.8	3.1
C18:1, Oleic, n-9	64.1	12.3
C18:2, Linoleic	56.2	17.8
C18:3, Linolenic	4.2	2.1
C20, Arachidic	0.4	0.1
C20:1	1.2	0.2
C20:2	1.4	0.2
C20:3, n-6	0.2	0.0
C20:4, Arachidonic, n-6	0.5	0.1
C22, Behenic	0.1	0.1
C22:5, Docosapentaenoic, n-3	0.2	0.0
Total (g)	190.7	43.1
Saturated (g)	60.2	10.1
Monounsaturated (g)	67.7	12.8
Polyunsaturated (g)	62.8	20.2
Saturated (% wt:wt)	31.6	23.5
Monounsaturated (% wt:wt)	35.5	29.7
Polyunsaturated (% wt:wt)	32.9	46.8
n6 (g)	57.0	17.9
n3 (g)	4.4	2.1
n6:n3 ratio	13.1	8.4
Trans fat (g)	0.0	0.0

CD: control diet (D12110704, Research Diets); g, gram; * HFS: high-fat/high-sucrose diet (D12451, Research Diets); wt, weight.
